# MRI-Based Radiomics of Basal Nuclei in Differentiating Idiopathic Parkinson’s Disease From Parkinsonian Variants of Multiple System Atrophy: A Susceptibility-Weighted Imaging Study

**DOI:** 10.3389/fnagi.2020.587250

**Published:** 2020-11-12

**Authors:** Huize Pang, Ziyang Yu, Renyuan Li, Huaguang Yang, Guoguang Fan

**Affiliations:** ^1^Department of Radiology, The first affiliated hospital of China Medical University, China Medical University, Shenyang, China; ^2^School of Medicine, Xiamen University, Xiamen, China; ^3^Interdisciplinary Institute of Neuroscience and Technology, School of Medicine, Zhejiang University, Hangzhou, China; ^4^The Affiliated Sir Run Run Shaw hospital, School of Medicine, Zhejiang University, Hangzhou, China

**Keywords:** idiopathic Parkinson’s disease, multiple system atrophy, radiomics, support vector machine, susceptibility weighted imaging

## Abstract

**Objectives:**

To investigate the value of MRI-based radiomic model based on the radiomic features of different basal nuclei in differentiating idiopathic Parkinson’s disease (IPD) from Parkinsonian variants of multiple system atrophy (MSA-P).

**Methods:**

Radiomics was applied to the 3T susceptibility- weighted imaging (SWI) from 102 MSA-P patients and 83 IPD patients (allocated to a training and a testing cohort, 7:3 ratio). The substantia nigra (SN), caudate nucleus (CN), putamen (PUT), globus pallidus (GP), red nucleus (RN), and subthalamic nucleus (STN) were manually segmented, and 396 features were extracted. After feature selection, support vector machine (SVM) was generated, and its predictive performance was calculated in both the training and testing cohorts using the area under receiver operating characteristic curve (AUC).

**Results:**

Seven radiomic features were selected from the PUT, by which the SVM classifier achieved the best diagnostic performance with an AUC of 0.867 in the training cohort and an AUC of 0.862 in the testing cohort. Furthermore, the combined model, which incorporating part III of the Parkinson’s Disease Rating Scale (UPDRSIII) scores into radiomic features of the PUT, further improved the diagnostic performance. However, radiomic features extracted from RN, SN, GP, CN, and STN had moderate to poor diagnostic performance, with AUC values that ranged from 0.610 to 0.788 in the training cohort and 0.583 to 0.766 in the testing cohort.

**Conclusion:**

Radiomic features derived from the PUT had optimal value in differentiating IPD from MSA-P. A combined radiomic model, which contained radiomic features of the PUT and UPDRSIII scores, further improved performance and may represent a promising tool for distinguishing between IPD and MSA-P.

## Introduction

Idiopathic Parkinson’s disease (IPD) and multiple system atrophy (MSA), especially Parkinsonian subtypes of MSA (MSA-P), are common neurodegenerative disorders that share similar Parkinsonism symptom (Ramli et al., 2015; Barbagallo et al., 2016). Although MSA-P may resemble IPD at the early stage, functional deterioration is more rapid, with moderate or transient dopaminergic responses, and contributes to a worse prognosis (Peeraully, 2014). Therefore, a development of an accurate diagnostic separation between IPD and MSA-P is of clinical significance.

More recently, increased attention has been paid to advanced magnetic resonance imaging (MRI) approaches to detect physiological mechanisms underlying PD and to distinguish IPD and MSA, and these approaches include resting-state functional MRI (Wang et al., 2017a), diffusion MRI (Hikishima et al., 2015), and voxel-based morphometry (Peran et al., 2018). However, these approaches are not generalized to clinical practice due to a lack of consistent results and their time-consuming nature. Susceptibility-weighted-imaging (SWI) has been widely used in clinical practice due to its sensitivity in detecting iron depositions (Liu et al., 2015), since loss of dopaminergic neurons and abnormal iron accumulation are well established as pathophysiological hallmarks of Parkinsonism (Hare et al., 2017; Chen et al., 2019). Promising MR diagnostic biomarkers have been proposed to be useful for differentiating IPD from atypical Parkinsonism (AP) via SWI based on neurodegenerative patterns that underlie PD and AP (Meijer et al., 2016; Wang et al., 2017b). However, consistent recognition of MR biomarkers has been met with difficulty among radiologists, especially when iron deposition is too low to be detected at the early stage of the disease, offering limited support for clinical diagnostic criteria. “Swallow-tail” sign has been demonstrated to be a promising biomarker for differentiating between IPD patients and healthy control (HCs), but not for discriminating IPD from AP (Wang et al., 2017b). Similarly, a distributional pattern of posterolateral putaminal hypointensity on SWI has been reported to be a common finding in MSA-P patients (Sugiyama et al., 2015; Lee et al., 2019). In practice, however, the inner or medial subregion of the putaminal hypointensity can also be found in MSA-P patients. Other patients may lack typical signs on SWI due to the relatively short period of disease. Additionally, tissue-specific physiological patterns in iron concentrations have been proposed, with the highest concentrations found in different basal nuclei [i.e., putamen (PUT), globus pallidus (GP), caudate nucleus (CN), and red nucleus (RN)] in patients with neurodegenerative diseases, which may provide valuable information for differential diagnoses (Han et al., 2013; Shahmaei et al., 2019). However, no single basal nucleus has been shown to completely distinguish between Parkinsonian disorders. On these premises, the potential of different basal nuclei in differentiating IPD from MSA-P requires further exploration.

Radiomics, which includes promising approaches that incorporate advanced quantification and classification methodologies, offers a complementary tool to existing radiological practices by extracting quantitative medical imaging features based on machine learning algorithms. A previous study has shown that radiomics offers important advantages for cancer diagnosis, grading, heterogeneity, and prognosis (Park et al., 2018). At present, there is growing interest in the potential of radiomics to aid in the development of non-invasive biomarkers in neurodegenerative diseases, such as PD and Alzheimer’s Disease (AD) (Feng et al., 2018; Cheng et al., 2019). However, the potential of radiomic analysis based on basal nuclei for distinguishing between PD and MSA-P on SWI has not yet been assessed.

Hence, in the present study, we investigated the most valuable nuclei for potentially enabling differential diagnosis of IPD and MSA-P based on a non-invasive radiomic model on SWI.

## Materials and Methods

### Participants

This investigation was approved by the Institutional Review Board of China Medical University, and written inform consent was obtained from all subjects. The IPD patients were diagnosed on the basis of the diagnostic criteria of the UK PD Society Brain Bank (Hughes et al., 1992). The MSA patients met the criteria for “probable MSA” via the second- consensus clinical criteria (Gilman et al., 2008). The exclusion criteria were as follows: (1) a history of cerebrovascular disease, brain tumor, or neurological surgery; (2) a history of substance abuse or alcohol dependence; (3) systemic diseases such as anemia and diabetes mellitus; (4) psychiatric disorders or co-occurring neurological illness; or (5) contraindications to an MRI examination. Following inclusion and exclusion criteria, 185 patients including 83 IPD patients and 102 MSA-P patients were recruited from the Department of Neurology between September 2016 and March 2019. The patients were randomly allocated to either the training (70%) or testing (30%) cohort, with stratified sampling. Movement disorders and the cognitive conditions of patients were assessed by part III of Parkinson’s Disease Rating Scale (UPDRSIII) and Montreal Cognitive Assessment (MoCA), respectively.

### MRI Acquisition

Magnetic resonance imaging scans were conducted on a 3.0T MRI scanner (Magnetom Verio, Siemens, Erlangen, Germany) with a 32-channel head coil. Based on 3D-FLASH T2WI sequence, the SWI data were obtained parallel to the anterior commissure-posterior commissure (AC-PC) plane, with the following parameters: repetition time/echo time = 27/20 ms; slice number = 64; slice thickness = 0.8 mm; flip angle = 15°; filed of view = 230 mm × 172.5 mm; matrix size = 182 × 256; and voxel size = 0.9 mm × 0.9 mm × 0.8 mm. All data were derived from one scanner and used the same MR parameters.

### Image Segmentation

**FIGURE 1:**
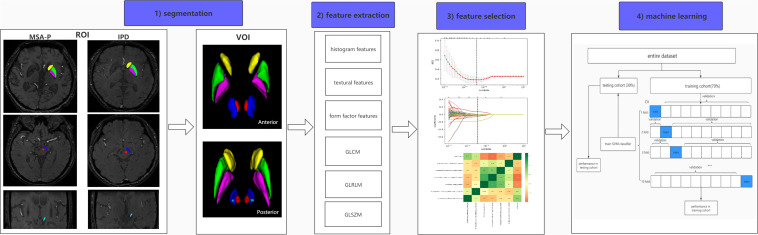
Workflow of radiomic analysis. (1) Regions of the CN (yellow), PUT (green), GP (purple), SN (blue), RN (red), and STN (light blue) were segmented slice by slice to generate volumes of interest (VOIs) in IPD and MSA-P patients. (2) Six kinds of radiomic features were extracted via AK software. (3) A combined feature-selection procedure was applied in the training cohort, which contained *t* tests, least absolute shrinkage and selection operator (LASSO), and Spearman correlation analysis. (4) The SVM classifier was constructed by ten-fold cross validation with 10 repetition in the training cohort, and the final diagnostic performance was evaluated in both training and testing cohorts.

Pathological studies have demonstrated that abnormal iron levels in a series of nigral and extranigral regions should be considered as candidate biomarkers to differentiate IPD from AP and controls, including the PUT, CN, GP, RN, subthalamic nuclei (STN), and substantia nigra (SN). On the basis of these studies, the selection of regions of interest (ROIs) was confirmed (Dickson, 2012; Mazzucchi et al., 2019). Considering that iron distribution is heterogenous, ROIs were drawn on the continuous layers to obtain volumes of interest (VOIs). Manual segmentation of the basal nuclei was carried out using ITK-SNAP (V3.4.0)^1^ according to the continuous anatomic structures with boundary voxels excluded. SN, CN, PUT, GP, and RN were segmented via axial-magnitude imaging, whereas STN was segmented via coronal-magnitude imaging by a neuroradiologist who was blinded to the clinical information (HP, with 5 years of experience in neuroimaging diagnoses). All segmentations were confirmed by a senior neuroradiologist (GF, with more than 20 years of experience in neuroimaging diagnosis). Figure 1 presents the workflow of the present study.

### Feature Extraction

Firstly, normalization (z-score transformation) was performed on the imaging data in order to avoid heterogeneity bias. In total, 396 features were ultimately extracted, including 41 histogram features, 72 statistics-based texture features, 10 form factor features, 82 gray-level co-occurrence matrix (GLCM) features, 180 gray-level run length matrix (GLRLM) features, and 11 gray-level size zone matrix (GLSZM) features. AK software (Artificial Intelligence Kit; version V3.2.0; GE Healthcare, China, Shanghai) was used for feature extraction. The details of radiomic feature extraction are presented in Supplementary Table S1.

### Feature Selection

To avoid model overfitting, the following steps were integrated in feature selection procedure in the training cohort: First, two sample *t* tests with a false-discovery rate (FDR) correction were applied to select features. Features were considered important at FDR-cor. *p* < 0.05. Subsequently, the least absolute shrinkage and selection operator (LASSO) regression was fitted to construct a subset of optimal features from the high-dimensional radiomic features using ten-fold cross-validation. Finally, Spearman’s rank correlation was employed for analyzing the correlation between the remaining non-zero radiomic features. The association was considered to be statistically significant when the absolute value of the coefficient was ≥0.8 and the *p* value was <0.05, thus excluding one of them according to their coefficients. The above steps were calculated using MATLAB R2013b. The optimization parameters are listed in Supplementary Table S2.

### Support Vector Machine (SVM) Construction and Validation

With the selected features, support vector machine (SVM) model with a radial basis function (RBF) kernel was performed for data classifications, owing to its classification stability and favorable performance. SVM has the potential to differentiate Parkinsonian syndromes and predict disease progression (Castillo-Barnes et al., 2018). The SVM classification was constructed using ten-fold cross-validation with 10 repetition in the training cohort. The statistical significance of the balanced accuracy (ACC) was identified by a random permutation test (1,000 times). The performance of the SVM classifier was evaluated by the area under the curve (AUC) in receiver operating characteristic (ROC) analysis in both the training and testing cohort. The SVM was implemented in MATLAB using the LIBSVM3.21 toolkit^2^.

### Statistical Analysis

The Kolmogorov–Smirnov test (K–S test) was performed to test the normality of each distribution. Student’s *t* test, Mann–Whitney test, and Chi-square tests were used for demographic variables and the selected radiomic features. To evaluate the balanced ACC, a permutation test (1,000 times) was also performed. *p* < 0.05 was deemed to be statistically significant. Statistical analyses were carried out using SPSS22.0 (SPSS Inc., Chicago, IL, United States) and MATLAB R 2013b.

## Results

### Demographic Characteristics

A total of 185 patients, including 83 IPD and 102 MSA-P patients, were included in the present study. There were no significant differences in age, gender, disease duration, or MoCA score between the IPD and MSA-P patients in both the training and testing cohorts. The MSA group showed slightly higher UPDRSIII scores compared with those in the IPD group in the training cohorts. However, there was no significant difference in UPDRSIII scores between the IPD and MSA-P groups in the testing cohorts (Table 1).

**TABLE 1:** Demographic characteristics of IPD and MSA-P patients in the training and testing cohorts.

Characteristics (mean ± SD)	Training cohort		*p*-value	Testing cohort		*p*-value
			
	IPD (*n* = 58)	MSA-P (*n* = 71)		IPD (*n* = 25)	MSA-P (*n* = 31)	
Age (y)	62.00 ± 7.55	64.44 ± 8.07	0.080	64.16 ± 6.52	62.48 ± 7.97	0.391
Gender (male/female)	28/30	37/34	0.665	12/13	15/16	0.977
Disease duration	4.42 ± 2.09	3.86 ± 1.91	0.116	4.46 ± 2.03	3.92 ± 1.82	0.304
UPDISIII score	37.66 ± 10.98	42.07 ± 13.33	0.041*	37.12 ± 9.14	41.74 ± 11.38	0.098
MoCA	22.60 ± 3.97	22.46 ± 4.37	0.851	21.03 ± 4.10	21.80 ± 4.17	0.494

SD, standard deviation; MSA-P, multiple system atrophy-Parkinsonian type; IPD, idiopathic Parkinson’s disease; UPDRSIII, Unified Parkinson’s Disease Rating Scale; MoCA, Montreal Cognitive Assessment; * denotes statistical significance, *P* < 0.05.

### Feature Selection

A total of 396 radiomic features were initially extracted from each basal nucleus. After performing *t* tests with FDR correction, the following significant features was selected: RN: four features; SN: 123 features; PUT: 69 features; GP: 138 features; CN:10 features; and STN: 22 features. Next, based on LASSO regression, the remaining features were as follows: three features (RN), 22 features (SN), seven features (PUT), 12 features (GP), three features (CN), and three features (STN). Finally, the most stable radiomic features were identified accordingly, as follows: RN: three features; SN: 16 features; PUT: seven features, GP: 12 features; CN: three features; and STN: two features. The details of the selected features in each basal nucleus are listed in Supplementary Table S3.

### Performances of Classifiers

**FIGURE 2:**
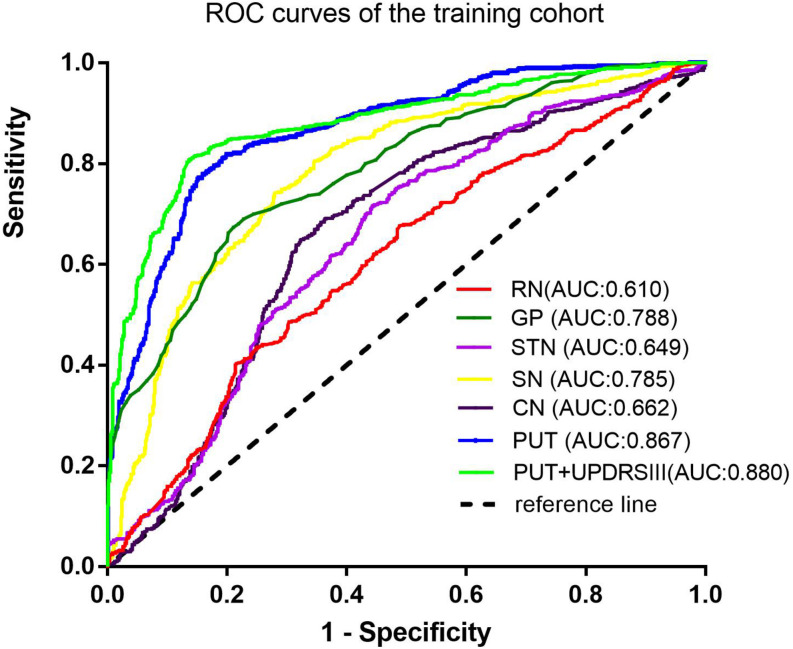
Receiver-operating characteristic (ROC) curves of the SVM model in the training cohort.

**FIGURE 3:**
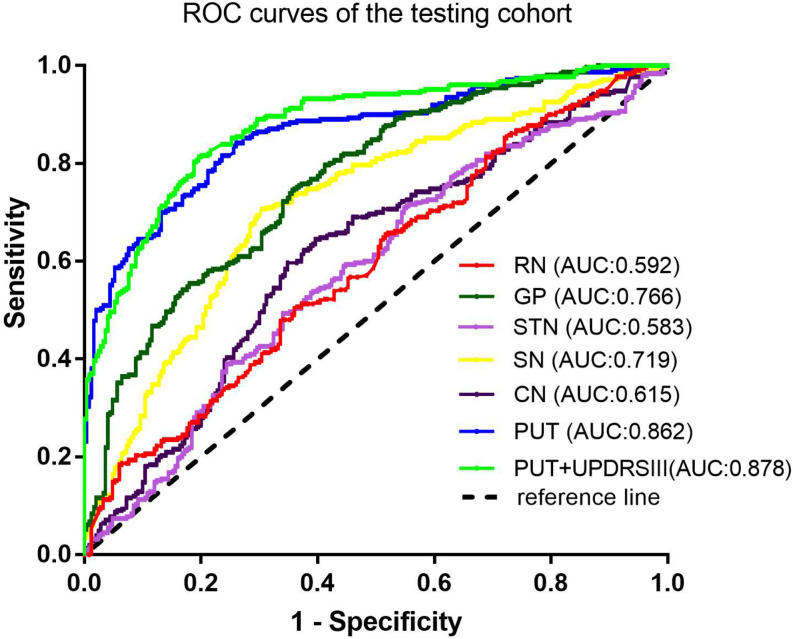
Receiver-operating characteristic (ROC) curves of the SVM model in the testing cohort.

Among the different basal nuclei, the SVM classifier showed the highest AUC using radiomic features extracted from the PUT, with an AUC of 0.867 for the training cohort and 0.862 for the testing cohort. In addition, the combined model, which added UPDRSIII scores to the radiomic model of the PUT, exhibited further improved classification performance. However, the SVM performances based on radiomic features of the GP, SN, RN, CN, and STN were moderate to poor, with AUC values ranging from 0.610 to 0.788 for the training cohort and from 0.583 to 0.766 for the testing cohort (Table 2 and Figures 2, 3).

**TABLE 2:** SVM classifier performance of each basal nucleus and the combined model in the training and testing cohorts.

Basal nucleus	Training cohort				*P(permut.)*	Testing cohort				*P(permut.)*
			
	Balanced-ACC	Sen (95%CI)	Spec (95%CI)	AUC (95%CI)		Balanced-ACC	Sen (95%CI)	Spec (95%CI)	AUC (95%CI)	
RN	0.595	0.512 (0.477, 0.551)	0.677 (0.634, 0.712)	0.610 (0.579, 0.641)	<0.01**	0.572	0.503 (0.446, 0.560)	0.640 (0.577, 0.700)	0.592 (0.545, 0.639)	<0.01**
SN	0.732	0.742 (0.708, 0.774)	0.721 (0.682, 0.757)	0.785 (0.759, 0.810)	<0.05*	0.702	0.707 (0.652, 0.757)	0.696 (0.635, 0.752)	0.719 (0.676, 0.761)	<0.01**
PUT	0.810	0.818 (0.788, 0.846)	0.802 (0.767, 0.833)	0.867 (0.847, 0.886)	<0.001***	0.791	0.842 (0.797, 0.881)	0.740 (0.681, 0.793)	0.862 (0.832, 0.892)	<0.001***
GP	0.731	0.690 (0.655, 0.724)	0.772 (0.736, 0.806)	0.788 (0.764, 0.812)	<0.001***	0.695	0.745 (0.693, 0.793)	0.644 (0.581, 0.703)	0.766 (0.727, 0.805)	<0.01**
CN	0.664	0.689 (0.653, 0.723)	0.638 (0.600, 0.677)	0.662 (0.631, 0.693)	<0.001***	0.626	0.645 (0.590, 0.698)	0.606 (0.540, 0.665)	0.615 (0.568, 0.662)	<0.01**
STN	0.636	0.718 (0.684, 0.751)	0.553 (0.512, 0.594)	0.649 (0.618, 0.679)	<0.01**	0.581	0.710 (0.656, 0.760)	0.452 (0.389, 0.516)	0.583 (0.535, 0.630)	<0.01**
PUT +	0.836	0.814 (0.784, 0.842)	0.857 (0.826, 0.884)	0.880 (0.861, 0.898)	<0.001***	0.809	0.813 (0.765, 0.855)	0.804 (0.749, 0.851)	0.878 (0.849, 0.906)	<0.001***

AUC, area under the receiver operator curve; RN, red nucleus; SN, substantia nucleus; PUT, putamen; GP, globus pallidus; CN, caudate nucleus; STN, subthalamic nucleus; Acc, accuracy; Sen, sensitivity; Spec, specificity; AUC, area under curve; CI, confidence interval. *denotes statistical significance, *P* < 0.05; **denotes statistical significance, *P* < 0.01; ***denotes statistical significance, *P* < 0.001.

### Representative Radiomic Feature Analysis in the PUT

After assembling the feature selection procedure, seven representative radiomic features were identified in the PUT, which included one histogram feature, one textural parameter, four GLCM features, and one GLRLM feature. The details of the representative radiomic features of the PUT are shown in Table 3.

**TABLE 3:** Statistical analysis of the selected radiomic features derived from the putamen.

Feature type-feature name	IPD	MSA-P	Stat/adjusted *P*-value
Histogram feature-Std Deviance	26.512 ± 5.337	34.949 ± 11.898	−5.001/<0.001***
Textural feature-Correlation_angle0_offset1	1.206E- 3 ± 7.177E-4	9.030E-4 ± 4.873E-4	2.849/<0.01**
GLCM feature-GLCMEntropv_AllDirection_offset7_SD	0.400 ± 0.353	0.769 ± 0.873	−3.026/<0.01**
GLCM feature-HaralickCorrelation_Alldirection_offset4	4.604E9 ± 2.821E9	1.829E9 ± 2.012E9	6.508/<0.001***
GLCM feature-InverseDifferenceMoment_angle0_offset7	0.045 ± 0.023	0.033 ± 0.017	3.377/<0.001***
GLCM feature-InverseDifferenceMoment_anglel35_offset7	0.043 ± 0.016	0.030 ± 0.016	4.552/<0.001***
GLRLM feature-RunLengtliNonuniformity_AllDirection_offset 4_SD	309.990 ± 120.300	400.501 ± 129.660	−4.073/<0.001***

MSA-P, multiple system atrophy-Parkinsonian type; IPD, idiopathic Parkinson’s disease; **denotes statistical significance, *P* < 0.01; ***denotes statistical significance, *P* < 0.001.

The histogram feature of the standard deviation (26.512 ± 5.337 in IPD; 34.949 ± 11.898 in MSA-P, *p* < 0.001) in MSA-P patients was higher than that in IPD patients. The textural feature- correlation (1.206E-3 ± 7.177E-4 in IPD, 9.030E-4 ± 4.873E-4 in MSA-P, *p* < 0.01) was lower in MSA-P patients, compared with that in IPD patients. As for GLCM features, the value of GLCM Entropy (0.400 ± 0.353 in IPD, 0.769 ± 0.873 in MSA-P, *p* < 0.01) was found to be comparatively higher in MSA-P patients. Furthermore, the Haralick correlation (4.604E9 ± 2.821E9 in IPD, 1.829E9 ± 2.012E9 in MSA-P, *p* < 0.001) and inverse difference moment (0.045 ± 0.023 in IPD, 0.033 ± 0.017 in MSA-P, *p* < 0.001) were higher in IPD patients compared with those in MSA-P patients. Meanwhile, the GLRLM- run length non-uniformity (309.990 ± 120.300 in IPD; 400.501 ± 129.660 in MSA-P; *p* < 0.001) in MSA-P patients was higher than that in IPD patients.

## Discussion

Multiple system atrophy, especially MSA-P variants, may mimic IPD particularly at the initial stage of the disease, as both MSA and IPD present with Parkinsonism (Peeraully, 2014; Ramli et al., 2015; Barbagallo et al., 2016). It is important to differentiate between IPD and MSA-P; however, it remains difficult to distinguish between these two neurodegenerative diseases via conventional MRI. Our present study is the first to build an SVM classifier using radiomic features derived from basal nuclei on SWI to differentiate IPD from MSA-P. We found that, based on the radiomic features derived from the PUT, the SVM classifier showed the best performance in differentiating IPD from MSA-P compared with that of the other basal nuclei. Furthermore, a combined model, which added UPDRSIII scores into the radiomic model of the PUT, further improved the classifier performance. However, radiomic features extracted from SN, GP, RN, STN, and CN showed only moderate to poor differential-diagnostic performances.

In the present study, the selected radiomic features in the PUT, extracted from magnitude imaging, consisted of one histogram parameter, one textural parameter, four GLCM parameters, and one GLRLM parameter, all of which revealed iron deposition patterns by reflecting higher-order imaging patterns and capturing mineable imaging heterogeneity. The histogram parameters described the statistical distribution of the voxel intensities within the established ROI. The histogram parameter- standard deviation is used to quantify the amount of variation or dispersion within an ROI. Our study found that the standard deviation was higher in MSA-P patients than in IPD patients, indicating the dispersed signal of the PUT on SWI in MSA-P patients, which was mainly attributed to marginal iron deposition. The textural parameter- correlation depicts the similarity of the gray levels in neighboring pixels. A higher correlation indicates a more homogeneous signal throughout the entire basal nuclei, which was found in IPD patients in the present study. The GLCM entropy is the measure of randomness of the intensities of images and indicates the amount of information in the image. The value of the GLCM entropy was found to be higher in MSA-P patients compared to that in IPD patients in the present study. The GLCM parameters- Haralick correlation and inverse difference moment describe the degree of similarity of the gray level in a row or column direction, and the local homogeneity of the image, respectively. These parameters were found to be lower in MSA-P patients than in IPD patients, suggesting heterogeneous iron decomposition of the PUT in MSA-P patients. GLRLM parameters measure how many pixels of a given gray value occur in a sequence in a given direction. The GLRLM parameter- run-length non-uniformity measures the similarity of run lengths. The value of run-length non-uniformity was found to be lower in IPD than that in MSA-P patients. This reflected the fact that iron accumulation of the PUT was more complex in MSA-P patients. A growing body of evidence has suggested that a pattern of putaminal hypointensity from lateral to medial on SWI is a specific feature in MSA-P patients (Gupta et al., 2010; Han et al., 2013; Shahmaei et al., 2019). Furthermore, uniform low-signal intensity throughout the PUT on SWI is thought to be non-specific and to accompany the normal aging process (Lee and Lee, 2019). As a result, the heterogeneity of the PUT on SWI was higher in patients with MSA-P than in IPD patients, which was in accordance with the results of our radiomic features. In this context, radiomic features may be served as an objective approach to assess the spatial distribution of iron deposition in the PUT, and may potentially applicable in clinical practice.

Furthermore, on the basis of these contributive features, we built an SVM classifier to enable an automated distinction between IPD and MSA-P patients. SVM, a method of machine learning, has been applied to neurodegenerative diseases based on the role of the basal nuclei. Chen et al. (2020) used morphologies of thalamic subnuclei as inputs to train the SVM and achieved a high accuracy of 95% in PD diagnosis. In another study, an SVM model was established using radiomic features extracted from Nigrosome-1, which yielded favorable results in terms of an AUC of 0.96 (Cheng et al., 2019). However, only a single basal nucleus was included in their analysis. Furthermore, few studies have focused on the differential diagnosis of PD and MSA-P. Hence, in our present study, we explored the power of differential diagnosis of SVM classifiers based on radiomic features extracted from six different basal nuclei and obtained an AUC of 0.867 in the training cohort and 0.862 in the testing cohort from PUT, implying its potential value in clinical applications.

A combined SVM model, which incorporated radiomic features and UPDRSIII score, was built on the basis of radiomic model extracted from the PUT in our present study. We found that the combined SVM model outperformed the radiomic SVM model. Considering that both MSA-P and IPD patients exhibit Parkinsonian symptoms, we used UPDRSIII scores to assess motor dysfunction as previously reported (Metman et al., 2004). MSA was associated with more rapidly progressive disease course; thus, UPDRSIII scores may be higher in MSA patients, especially at the initial stage of the disease (Krismer et al., 2019). Some previous studies have found that there is a correlation between R2^∗^ values and the degree of clinical manifestations in progressive supranuclear palsy (PSP) patients, which suggests that the severity of rigidity and tremors is associated with iron-related pathologies (Lee et al., 2017). The radiomic features captured imaging heterogeneity by revealing higher-order imaging patterns, which conferred better performance compared to that of conventional approaches. The heterogeneity of SWI is mainly influenced by iron decomposition occurring locally in PD. Thus, UPDRSIII scores may serve as additional radiomic features to unveil heterogeneity patterns within images. Incorporating UPDRSIII scores into a developed radiomic model allows for more information; thus, the combined model may provide further benefit for diagnostic performance. This also indicates that a combination of radiological examinations and clinical symptoms is necessary for making clinical decisions. However, in view of the instability and subjectivity of clinical scales, the diagnostic performance of UPDRSIII scores combined with radiomic features warrants further investigation.

In our present study, the performances of the SVM classifier based on radiomic features extracted from GP, SN, CN, RN, and STN showed moderate to poor performance in terms of differential diagnosis. According to previous studies, an absence of dorsolateral nigral hyperintensity on MRI indicating a valuable marker for identifying IPD and can also be found in the majority of AP patients, preventing differential diagnosis among the IPD and AP patients (Bae et al., 2016; Mahlknecht et al., 2017). However, other researchers have suggested that the absence of “swallow-tail” sign may have potential in differentiating PD from MSA patients (Wang et al., 2017b). It was reported that the presence of “swallow-tail” sign was more prevalent in cerebellar subtypes of MSA (MSA-C) in contrast to MSA-P patients (Reiter et al., 2015). Thus, this discrepancy might be due to the failure of subtyping MSA patients. Our study also supported the notion that the pathogenesis of SN in MSA may be associated with iron deposition and reduced neuromelanin, similar to features found in IPD patients. Recent SWI studies have demonstrated that the susceptibilities of the RN and GP are higher in PSP patients compared with those in IPD or MSA patients, as well as controls (Han et al., 2013; Sjostrom et al., 2017). However, the RN and GP were not capable of differentiating between MSA from IPD patients in our study. Furthermore, the CN has been reported to be have no statistical differences in iron content between PD and AP patients (Han et al., 2013; Sjostrom et al., 2017; Mazzucchi et al., 2019). Similarly, in our present study, the radiomic features extracted from the RN, GP, and CN showed moderate to poor differential diagnostic values. However, Mazzucchi et al. (2019) found that the STN might be useful for differentiating MSA from PD. This discrepancy may be due to the small size of the STN and difficulty in its visualization on SWI.

There were some limitations to our present study. First, the SVM classifier was built based on ROIs that were manually drawn on SWI, which represents a tedious and inconvenient process for clinical application. Therefore, development of automated segmentation methods is needed in the future. Second, only six representative basal nuclei and corresponding SVM classifiers were included in our present study. Hence, more basal nuclei and machine-learning methods should be taken into consideration in future studies. Third, compared to that of SWI, quantitative susceptibility mapping (QSM) and R2^∗^ mapping tend to be more sensitive quantitative methods for estimating iron deposition in PD patients (Liu et al., 2015; Chen et al., 2019; Mazzucchi et al., 2019). Therefore, further research should be performed that relates radiomic features to quantitative iron contents within different basal nuclei.

In conclusion, we found that radiomic features derived from the PUT exhibited the best performance in differentiating IPD from MSA-P patients. Furthermore, a combined radiomic model containing radiomic features of the PUT and UPDRSIII scores further improved diagnostic performance and may be useful as a diagnostic tool distinguishing between IPD and MSA-P.

## Data Availability Statement

The datasets generated for this study are available on request to the corresponding author.

## Ethics Statement

The studies involving human participants were reviewed and approved by this investigation was approved by the Institutional Review Board of China Medical University. The patients/participants provided their written informed consent to participate in this study.

## Author Contributions

GF: the conception and design of the study and SWI data confirmation. HP: SWI data segmentation, analysis and interpretation of data, and drafting the article. ZY and RL: analysis of data and statistical analysis. HY: acquisition of data. All authors contributed to the article and approved the submitted version.

## Conflict of Interest

The authors declare that the research was conducted in the absence of any commercial or financial relationships that could be construed as a potential conflict of interest.

## Footnotes

1 http://www.itksnap.org

2 https://www.csie.ntu.edu.tw/~cjlin/libsvm/

## Abbreviations

AP: Atypical Parkinsonism
AUC: Area under the curve
CN: Caudate nucleus
GP: Globus pallidus
IPD: Idiopathic Parkinson’s disease
LASSO: The least absolute shrinkage and selection operator
MSA-P: Parkinsonian variant of multiple system atrophy
MRI: Magnetic resonance imaging
MoCA: Montreal Cognitive Assessment
PUT: Putamen
PSP: Progressive supranuclear palsy
ROC: Receiver operating characteristic
RN: Red nucleus
SN: Substantia nigra
STN: Subthalamic nucleus
SWI: Susceptibility-weighted imaging
SVM: Support vector machine
UPDRSIII: Parkinson’s Disease Rating Scale III.

## References

[B1] Bae Y. J. Kim J. M. Kim E. Lee K. M. Kang S. Y. Park H. S. (2016). Loss of nigral hyperintensity on 3 tesla MRI of parkinsonism: comparison with 123I-FP-CIT SPECT. *Mov. Disord.* 31 684–692. 10.1002/mds.26584 26990970

[B2] Barbagallo G. Sierra-Pena M. Nemmi F. Traon A. P. Meissner W. G. Rascol O. (2016). Multimodal MRI assessment of nigro-striatal pathway in multiple system atrophy and parkinson disease. *Mov. Disord.* 31 325–324. 10.1002/mds.26471 26676922

[B3] Castillo-Barnes D. Ramirez J. Segovia F. Martinez-Murcia F. J. Salas-Gonzalez D. Gorriz J. M. (2018). Robust ensemble classification methodology for I123-ioflupane SPECT images and multiple heterogeneous biomarkers in the diagnosis of parkinson’s disease. *Front. Neuroinform.* 12:53. 10.3389/fninf.2018.00053 30154711 PMC6102321

[B4] Chen Q. Chen Y. Zhang Y. Wang F. Yu H. Zhang C. (2019). Iron deposition in parkinson’s disease by quantitative susceptibility mapping. *BMC Neurosci.* 20:23. 10.1186/s12868-019-0505-9 31117957 PMC6532252

[B5] Chen Y. Zhu G. Liu D. Liu Y. Yuan T. Zhang X. (2020). The morphology of thalamic subnuclei in parkinson’s disease and the effects of machine learning on disease diagnosis and clinical evaluation. *J. Neuro. Sci.* 411:116721. 10.1016/j.jns.2020.116721 32058183

[B6] Cheng Z. Zhang J. He N. Li Y. Wen Y. Xu H. (2019). Radiomic features of the nigrosome-1 region of the the substantia nigra: using quantitative susceptibility mapping to assist the diagnosis of idiopathic parkinson’s disease. *Front. Aging. Neurosci.* 11:167. 10.3389/fnagi.2019.00167 31379555 PMC6648885

[B7] Dickson D. W. (2012). Parkinson’s disease and parkinsonism: neuropathology. *Cold Spring Harb. Perspect. Med.* 1:2. 10.1101/cshperspect.a009258 22908195 PMC3405828

[B8] Feng Q. Chen Y. Liao Z. Jiang H. Mao D. Wang M. (2018). Corpus callosum radiomics-based classification model in alzheimer’s disease: a case-control study. *Front. Neurol.* 9:618. 10.3389/fneur.2018.00618 30093881 PMC6070743

[B9] Gilman S. Wenning G. K. Low P. A. Brooks D. J. Mathias C. J. Trojanowski J. Q. (2008). Second consensus statement on the diagnosis of multiple system atrophy. *Neurology* 71 670–676. 10.1212/01.wnl.0000324625.00404.15 18725592 PMC2676993

[B10] Gupta D. Saini J. Kesavadas C. Sarma P. S. Kishore A. (2010). Utility of susceptibility-weighted MRI in differentiating parkinson’s disease and atypical parkinsonism. *Neuroradiology* 52 1087–1094. 10.1007/s00234-010-0677-6 20358367

[B11] Han Y. H. Lee J. H. Kang B. M. Mun C. W. Baik S. K. Shin Y. I. (2013). Topographical differences of brain iron deposition between progressive supranuclear palsy and parkinsonian variant multiple system atrophy. *J. Neurol. Sci.* 325 29–35. 10.1016/j.jns.2012.11.009 23260321

[B12] Hare D. J. Cardoso B. R. Raven E. P. Double K. L. Finkelstein D. I. Szymlek-Gay E. A. (2017). Excessive early-life dietary exposure: a potential source of elevated brain iron and a risk factor for parkinson’s disease. *NPJ Parkinsons Dis.* 3:1. 10.1038/s41531-016-0004-y 28649601 PMC5460187

[B13] Hikishima K. Ando K. Yano R. Kawai K. Komaki Y. Inoue T. (2015). Parkinson disease: diffusion MR imaging to detect nigrostriatal pathway loss in a marmoset model treated with 1-methyl-4-phenyl-1,2,3,6-tetrahydropyridine. *Radiology* 275 430–437. 10.1148/radiol.14140601 25602507

[B14] Hughes A. J. Daniel S. E. Kilford L. Lees A. J. (1992). Accuracy of clinical diagnosis of idiopathic parkinson’s disease: a clinic-pathological study of 100 cases. *J. Neurol. Neurosurg. Psychiatry.* 55 181–184. 10.1136/jnnp.55.3.181 1564476 PMC1014720

[B15] Krismer F. Seppi K. Gobel G. Steiger R. Zucal I. Boesch S. (2019). Morphometric MRI profiles of multiple system atrophy variants and implications for differential diagnosis. *Mov. Disord.* 34 1041–1048. 10.1002/mds.27669 30919495 PMC6767501

[B16] Lee J. H. Lee M. S. (2019). Brain iron accumulation in atypical parkinsonian syndromes: in vivo MRI evidence for distinctive patterns. *Front. Neurol.* 10:74. 10.3389/fneur.2019.00074 30809185 PMC6379317

[B17] Lee M. J. Kim T. H. Kim S. J. Kim B. K. Mun C. W. Lee J. H. (2019). Quantitative validation of a visual rating scale for defining high-iron putamen in patients with multiple system atrophy. *Front. Neurol.* 10:1014. 10.3389/fneur.2019.01014 31616365 PMC6763953

[B18] Lee S. H. Lyoo C. H. Ahn S. J. Rinne J. O. Lee M. S. (2017). Brain regional iron contents in progressive supranuclear palsy. *Parkinsonism Relat. Disord.* 45 28–32. 10.1016/j.parkreldis.2017.09.020 28982612

[B19] Liu C. Li W. Tong K. A. Yeom K. W. Kuzminski S. (2015). Susceptibility-weighted imaging and quantitative susceptibility mapping in the brain. *J. Magn. Reson. Imaging* 42 23–41. 10.1002/jmri.24768 25270052 PMC4406874

[B20] Mahlknecht P. Krismer F. Poewe W. Seppi K. (2017). Meta-analysis of dorsolateral nigral hyperintensity on magnetic resonance imaging as a marker for parkinson’s disease. *Mov. Disord.* 32 619–623. 10.1002/mds.26932 28151553

[B21] Mazzucchi S. Frosini D. Costaqli M. Del Prete E. Donatelli G. Cecchi P. (2019). Quantitative susceptibility mapping in atypical parkinsonisms. *Neuroimage Clin.* 24:101999. 10.1016/j.nicl.2019.101999 31539801 PMC6812245

[B22] Meijer F. J. Steens S. C. Van Rumund A. Van Cappellen van Walsum A. M. Kusters B. (2016). Nigrosome-1 on susceptibility weighted imaging to differentiate parkinson’s disease from atypical parkinsonism: an in vivo and ex vivo pilot study. *Pol. J. Radiol.* 81 363–369. 10.12659/PJR.897090 27559425 PMC4975367

[B23] Metman L. V. Myre B. Verwey N. Hassin-Baer S. Arzbaecher J. Sierens D. (2004). Test-retest reliability of UPDRSIII, dyskinesia scales, and time motor tests in patients with advanced parkinson’s disease: an argument against multiple baseline assessments. *Mov. Disord.* 19 1079–1084. 10.1002/mds.20101 15372601

[B24] Park H. Lim Y. Ko E. S. Cho H. H. Lee J. E. Han B. K. (2018). Radiomics signature on magnetic resonance imaging: association with disease- free survival in patients with invasive breast cancer. *Clin. Cancer. Res.* 24 4705–4714. 10.1158/1078-0432.CCR-17-3783 29914892

[B25] Peeraully T. (2014). Multiple system atrophy. *Semin. Neurol.* 34 174–181. 10.1055/s-0034-1381737 24963676

[B26] Peran P. Barbagallo G. Nemmi F. Sierra M. Galitzky M. Traon A. P. (2018). MRI supervised and unsupervised classification of Parkinson’s disease and multiple system atrophy. *Mov. Disord.* 33 600–608. 10.1002/mds.27307 29473662

[B27] Ramli N. Nair S. R. Ramli N. M. Lim S. Y. (2015). Differentiating multiple-system atrohy from parkinson’s disease. *Clin. Radiol.* 70 555–564. 10.1016/j.crad.2015.01.005 25752581

[B28] Reiter E. Mueller C. Pinter B. Krismer F. Scherfler C. Esterhammer R. (2015). Dorsolateral nigral hyperintensity on 3.0T susceptibility-weighted imaging in neurodegenerative Parkinsonism. *Mov. Disord.* 30 1068–1076. 10.1002/mds.26171 25773707

[B29] Shahmaei V. Faeghi F. Mohammdbeigi A. Hashemi H. Ashrafi F. (2019). Evaluation of iron deposition in brain basal ganglia of patients with parkinson’s disease using quantitative susceptibility mapping. *Eur. J. Radiol. Open* 6 169–174. 10.1016/j.ejro.2019.04.005 31065578 PMC6495059

[B30] Sjostrom H. Granberg T. Westman E. Svenningsson P. (2017). Quantitative susceptibility mapping differentiates between parkinsonian disorders. *Parkinsonism Relat. Disord.* 44 51–57. 10.1016/j.parkreldis.2017.08.029 28886909

[B31] Sugiyama A. Ito S. Suichi T. Sakurai T. Mukai H. Yokota H. (2015). Putaminal hypotensity on T2^∗^-weighted MR imaging is the most practically useful sign in diagnosing multiple system atrophy: a preliminary study. *J. Neurol. Sci.* 349 174–178. 10.1016/j.jns.2015.01.013 25619571

[B32] Wang N. Edmiston E. K. Luo X. Yang H. Chang M. Wang F. (2017a). Comparing abnormalities of amplitude of low-frequency fluctuations in multiple system atrophy and idiopathic parkinson’s disease measured with resting-state fMRI. *Psych. Res. Neuroimaging* 269 73–81. 10.1016/j.pscychresns.2017.09.002 28957750

[B33] Wang N. Yang H. Li C. Fan G. Luo X. (2017b). Using “swallow-tail” sign and putaminal hypointensity as biomarkers to distinguish multiple system atrophy from idiopathic parkinson’s disease: a susceptibility-weighted imaging study. *Eur. Radiol.* 27 3174–3180. 10.1007/s00330-017-4743-x 28105503

