# Supplementary material for: MRI-Based Radiomics of Basal Nuclei in Differentiating Idiopathic Parkinson’s Disease From Parkinsonian Variants of Multiple System Atrophy: A Susceptibility-Weighted Imaging Study
Source: Front Aging Neurosci. 2020 Nov 12;12:587250. doi: 10.3389/fnagi.2020.587250 (PMC7689200; doi:10.3389/fnagi.2020.587250)
Supplement: Supplementary file 1 [file Table_1.DOCX]

Supplementary Table 1. Six groups of radiomic features

| **Group** |
| --- |
| **1. Histogram features (n = 41)**  Energy; Entropy; Max Intensity; Min Intensity; Kurtosis; Mean Value; Mean Deviation; Median Intensity; Range; Root mean square; Skewness; Standard Deviation; Uniformity; Variance; Volume Count; Voxel Value Sum; Relative Deviation; Frequency Size; Quantile(0.025,0.25,0.5,0.75,0.975);Percentile(5,10,15,20,25,30,35,40,45,50,55,60,65,70,75,80,85,90,95)  **2. Statistics-based texural features (n =72)**  Correlation*; Inertia*; Cluster Shade*; Cluster Prominence*  **3. Form factor features (n = 10)**  Sphericity; Surface area; Compactness 1; Compactness 2; Maximum 3D diameter; Spherical disproportion; SurfaceVolumeRatio; Voxel Volume; Mesh Volume; One Voxel Volume  **4. GLCM (n = 82)**  GLCMenergy*; GLCMentropy*; Inverse Difference Moment*; Haralick features (Haralick correlation*; Angular Second Moment; Contrast; Hara Entropy; Hara Variance; Sum Average; Sum Variance; Sum Entropy; Difference Variance; Difference Entropy; InverseDifferenceMoment)  **5. GLRLM (n=180)**  ShortRunEmphasis*; LongRunEmphasis*; GreyLevelNon-uniformity*; RunLengthNon-uniformity*; LowGreyLevelRunEmphasis*; HighGreyLevelRunEmphasis*; ShortRunLowGrey Level Emphasis*; ShortRunHighGreyLevelEmphasis*; LongRunLowGreyLevel Emphasis*; LongRunHighGreyLevel Emphasis*  **6.GLSZM (n=11)**  Small Area Emphasis; Large Area Emphasis; Zone Percentage; Low Intensity Emphasis; High Intensity Emphasis; Low Intensity Small Area Emphasis; High Intensity Small Area Emphasis; Low Intensity Large Area Emphasis; High Intensity Large Area Emphasis; Intensity Variability; Size Zone Variability |

*:18directions:AllDirection_offset1,AllDirection_offest1_SD,AllDirection_offset4,AllDirection_offset4_SD,AllDirection_offset7,AllDirection_offset7_SD,angle0_offset1,angle0_offset4,angle0_offset7,angle45_offset1,angle45_offset4,angle45_offset7,angle90_offset1,angle90_offset4,angle90_offset7, angle135_offset1, angle135_offset4, angle135_offset7

Abbreviations: GLCM: grey-level co-occurrence matrix, GLRLM: grey-level run length matrix GLSZM: grey-level size zone matrix.
