# Supplementary material for: MRI-Based Radiomics of Basal Nuclei in Differentiating Idiopathic Parkinson’s Disease From Parkinsonian Variants of Multiple System Atrophy: A Susceptibility-Weighted Imaging Study
Source: Front Aging Neurosci. 2020 Nov 12;12:587250. doi: 10.3389/fnagi.2020.587250 (PMC7689200; doi:10.3389/fnagi.2020.587250)
Supplement: Supplementary file 2 [file Table_2.DOCX]

Supplementary Table 2. The optimization parameters of each basal nucleus and the combined model

| **Basal nucleus** | **Lambda** | **Best-c** | **Best-g** |
| --- | --- | --- | --- |
| RN | 0.001 | 0.926 | 0.100 |
| SN | 0.004 | 3.175 | 0.010 |
| PUT | 0.036 | 0.500 | 0.010 |
| GP | 0.024 | 3.175 | 0.005 |
| CN | 0.010 | 1.714 | 37.276 |
| STN | 0.036 | 0.500 | 2.683 |
| PUT+UPDRSIII | 0.036 | 0.680 | 0.005 |

Abbreviations: RN: red nucleus. SN: substantia nigra. PUT: putamen; GP: globus pallidus.CN: caudate. STN: subthalamic nucleus. UPDRSIII: part III of Parkinson’s Disease Rating Scale.
