# Supplementary material for: MRI-Based Radiomics of Basal Nuclei in Differentiating Idiopathic Parkinson’s Disease From Parkinsonian Variants of Multiple System Atrophy: A Susceptibility-Weighted Imaging Study
Source: Front Aging Neurosci. 2020 Nov 12;12:587250. doi: 10.3389/fnagi.2020.587250 (PMC7689200; doi:10.3389/fnagi.2020.587250)
Supplement: Supplementary file 3 [file Table_3.DOCX]

Supplementary Table 3. Radiomic features selected by feature selection procedure of each basal nucleus

| Basal nucleus | feature number | Selected features | formula |
| --- | --- | --- | --- |
| RN | 3 | GLCMEntropy_AllDirection_offset4_SD  GLCMEntropy_angle90_offset4  GLCMEntropy_angle90_offset7 | $-\sum_{i=1}^{N_{g}} \sum_{j=1}^{N_{g}} p(i,j){log}_{2}[p(i,j)]$  $-\sum_{i=1}^{N_{g}} \sum_{j=1}^{N_{g}} p(i,j){log}_{2}[p(i,j)]$  $-\sum_{i=1}^{N_{g}} \sum_{j=1}^{N_{g}} p(i,j){log}_{2}[p(i,j)]$ |
| SN | 16 | ClusterProminence_angle45_offset1  ClusterShade_AllDirection_offset1_SD  Correlation_AllDirection_offset4  Correlation_angle0_offset4  Correlation_angle90_offset1  GLCMEnergy_angle135_offset4  GLCMEntropy_angle135_offset7  HaralickCorrelation_angle0_offset4  HaralickCorrelation_angle135_offset1  Inertia_AllDirection_offset4_SD  Inertia_angle90_offset4  InverseDifferenceMoment_angle90_offset1  LongRunEmphasis_AllDirection_offset1  ShortRunEmphasis_AllDirection_offset7  ShortRunEmphasis_angle0_offset1  ShortRunLowGreyLevelEmphasis_angle0_offset4 | $\sum_{i=1}^{N_{g}} \sum_{j=1}^{N_{g}} \left[ i+j-\mu_{x}-\mu_{y} \right]^{4}p(i,j)$  $\sum_{i=1}^{N_{g}} \sum_{j=1}^{N_{g}} \left[ i+j-\mu_{x}-\mu_{y} \right]^{3}p(i,j)$  $\frac{\sum_{i=1}^{N_{g}} \sum_{j=1}^{N_{g}} ijp(i,j)-\mu_{x}\mu_{y}}{{}_{x}{}_{y}}$  $\frac{\sum_{i=1}^{N_{g}} \sum_{j=1}^{N_{g}} ijp(i,j)-\mu_{x}\mu_{y}}{{}_{x}{}_{y}}$  $\frac{\sum_{i=1}^{N_{g}} \sum_{j=1}^{N_{g}} ijp(i,j)-\mu_{x}\mu_{y}}{{}_{x}{}_{y}}$  $\sum_{i=1}^{N_{g}} \sum_{j=1}^{N_{g}} {[p(i,j)]}^{2}$  $-\sum_{i=1}^{N_{g}} \sum_{j=1}^{N_{g}} p(i,j){log}_{2}[p(i,j)]$  $\sum_{i,j} \frac{(i,j)g(i,j)-\mu_{t}^{2}}{{}_{t}^{2}}$  $\sum_{i,j} \frac{(i,j)g(i,j)-\mu_{t}^{2}}{{}_{t}^{2}}$  $\sum_{i,j} {((i-j)}^{2}g(i,j))$  $\sum_{i,j} {((i-j)}^{2}g(i,j))$  $\sum_{i=1}^{N_{g}} \sum_{j=1}^{N_{g}} \frac{p(i,j)}{1+\frac{\vert i-j\vert}{N_{g}}}$  $\frac{1}{n_{r}}\sum_{i=1}^{M} \sum_{j=1}^{N} p(i,j,)j^{2}$  $\frac{1}{n_{r}}\sum_{i=1}^{M} \sum_{j=1}^{N} \frac{p(i,j,)}{j^{2}}$  $\frac{1}{n_{r}}\sum_{i=1}^{M} \sum_{j=1}^{N} \frac{p(i,j,)}{j^{2}}$  $\frac{1}{n_{r}}\sum_{i=1}^{N} \sum_{j=1}^{M} \frac{p(i,j,)i^{2}}{j^{2}}$ |
| PUT | 7 | Std Deviance  Correlation_angle0 _offset1  GLCMEntropy_Alldirection_offset7_SD  HaralickCorrelation_Alldirection_offset4  InverseDifferenceMoment_angle0_offset7  InverseDifferenceMoment_angle135_offset7  RunLengthNonuniformity_AllDirection_offset4_SD | $\sqrt{\frac{1}{N}\sum_{i=1}^{N} {(x(i)-\bar{x)}}^{2}}$  $\frac{\sum_{i=1}^{N_{g}} \sum_{j=1}^{N_{g}} ijp(i,j)-\mu_{x}\mu_{y}}{{}_{x}{}_{y}}$  $-\sum_{i=1}^{N_{g}} \sum_{j=1}^{N_{g}} p(i,j){log}_{2}[p(i,j)]$  $\sum_{i,j} \frac{(i,j)g(i,j)-\mu_{t}^{2}}{{}_{t}^{2}}$  $\sum_{i=1}^{N_{g}} \sum_{j=1}^{N_{g}} \frac{p(i,j)}{1+\frac{\vert i-j\vert}{N_{g}}}$  $\sum_{i=1}^{N_{g}} \sum_{j=1}^{N_{g}} \frac{p(i,j)}{1+\frac{\vert i-j\vert}{N_{g}}}$  $\frac{1}{n_{r}}\sum_{j=1}^{N} (\sum_{i=1}^{M} {p(i,j,))}^{2}$ |
| GP | 12 | Histogram Energy  Correlation_angle135_offset7  HaralickCorrelation_AllDirection_offset7  InverseDifferenceMoment_AllDirection_offset4_SD  InverseDifferenceMoment_AllDirection_offset7_SD  InverseDifferenceMoment_angle0_offset7  InverseDifferenceMoment_angle45_offset7  InverseDifferenceMoment_angle90_offset4  Compactness1  ShortRunEmphasis_angle90_offset1  ShortRunEmphasis_angle0_offset7  RunLengthNonuniformity_AllDirection_offset7 | $\sum_{i}^{N} X{(i)}^{2}$  $\frac{\sum_{\boldsymbol{i=1}}^{\boldsymbol{N}_{\boldsymbol{g}}} \sum_{j=1}^{N_{g}} ijp(i,j)-\mu_{x}\mu_{y}}{{}_{\boldsymbol{x}}{}_{\boldsymbol{y}}}$  $\sum_{i,j} \frac{(i,j)g(i,j)-\mu_{t}^{2}}{{}_{t}^{2}}$  $\sum_{i=1}^{N_{g}} \sum_{j=1}^{N_{g}} \frac{p(i,j)}{1+\frac{\vert i-j\vert}{N_{g}}}$  $\sum_{i=1}^{N_{g}} \sum_{j=1}^{N_{g}} \frac{p(i,j)}{1+\frac{\vert i-j\vert}{N_{g}}}$  $\sum_{i=1}^{N_{g}} \sum_{j=1}^{N_{g}} \frac{p(i,j)}{1+\frac{\vert i-j\vert}{N_{g}}}$  $\sum_{i=1}^{N_{g}} \sum_{j=1}^{N_{g}} \frac{p(i,j)}{1+\frac{\vert i-j\vert}{N_{g}}}$  $\sum_{i=1}^{N_{g}} \sum_{j=1}^{N_{g}} \frac{p(i,j)}{1+\frac{\vert i-j\vert}{N_{g}}}$  $\frac{V}{{\sqrt{\pi}A}^{\frac{2}{3}}}$  $\frac{1}{n_{r}}\sum_{i=1}^{M} \sum_{j=1}^{N} \frac{p(i,j,)}{j^{2}}$  $\frac{1}{n_{r}}\sum_{i=1}^{M} \sum_{j=1}^{N} \frac{p(i,j,)}{j^{2}}$  $\frac{1}{n_{r}}\sum_{j=1}^{N} (\sum_{i=1}^{M} {p(i,j,))}^{2}$ |
| CN  STN | 3  2 | Range  GLCMEntropy_AllDirection_offset7_SD  HaralickCorrelation_AllDirection_offset1_SD  GLCMEntropy_angle45_offset4  RunLengthNonuniformity_AllDirection_offset1_SD | The range of intensity X  $-\sum_{i=1}^{N_{g}} \sum_{j=1}^{N_{g}} p(i,j){log}_{2}[p(i,j)]$  $\sum_{i,j} \frac{(i,j)g(i,j)-\mu_{t}^{2}}{{}_{t}^{2}}$  $-\sum_{i=1}^{N_{g}} \sum_{j=1}^{N_{g}} p(i,j){log}_{2}[p(i,j)]$  $\frac{1}{n_{r}}\sum_{j=1}^{N} (\sum_{i=1}^{M} {p(i,j,))}^{2}$ |
|  |  |  |  |

Abbreviations: RN, red nucleus; SN, substantia nucleus; PUT, putamen; GP, globus pallidus; CN, caudate nucleus; STN, subthalamic nucleus; GLCM, gray level co-occurrence matrix
